# uPAR-Targeting Cytotoxic Antibody–Drug Conjugates Selectively Deplete Proinflammatory Myeloid Cells for Autoimmune Indications

**DOI:** 10.3390/cells15090803

**Published:** 2026-04-29

**Authors:** Handan Xiang, Grace Pham Mortenson, Simon B. Lang, Sriram Jakkaraju, Anirudh Chirala, Yimin Zhu, Mengxuan Jia, Jianzhong Wen, Ying Chen, Arjun Baghela, Yen-Cheng Chen, Marc A. Sze, Laxminarayan G. Hegde, Jie Zhang-Hoover, Aarron Willingham, Masahisa Handa, An Chi, Gretchen A. Baltus, Rajesh V. Kamath, Marc C. Levesque, Elisabeth H. Vollmann

**Affiliations:** 1 Discovery Immunology, Merck & Co., Inc., Rahway, NJ 07065, USA; 2 Discovery Biologics, Merck & Co., Inc., Rahway, NJ 07065, USA; grace.mortenson@merck.com (G.P.M.); sriram.jakkaraju@merck.com (S.J.); masahisa.handa@merck.com (M.H.); 3 Discovery Chemistry, Merck & Co., Inc., Rahway, NJ 07065, USA; 4 Quantitative Biosciences, Merck & Co., Inc., Rahway, NJ 07065, USA; 5 Pharmacokinetics, Dynamics, Metabolism and Bioanalytics, Merck & Co., Inc., Rahway, NJ 07065, USA; 6 Data, AI and Genome Sciences, Merck & Co., Inc., Rahway, NJ 07065, USA; 7 Chemical Biology, Merck & Co., Inc., Rahway, NJ 07065, USA

**Keywords:** rheumatoid arthritis, proinflammatory myeloid cells, uPAR, antibody–drug conjugates (ADCs), BCL-2 family inhibitors, cell death

## Abstract

**Highlights:**

**What are the main findings?**

- Cytotoxic uPAR–BCL-2 inhibitor conjugates selectively deplete uPAR^high^-expressing primary human inflammatory monocytes and macrophages and reduce cytokine production.
- uPAR–MMAF conjugates deplete uPAR^high^ CD11b^+^F4/80^+^ macrophages in an air pouch model.

**What are the implications of the main findings?**

- uPAR may serve as a cell-surface marker to target and eliminate proinflammatory monocytes and macrophages.
- Immunology ADCs can be developed to eliminate disease-associated cell subsets in autoimmune indications.

**Abstract:**

Rheumatoid arthritis (RA) is an autoimmune disorder characterized by synovial inflammation and progressive joint destruction. There is no cure, and patient responses to current therapies vary, reflecting underlying pathogenic heterogeneity. Leveraging single-cell RNA sequencing (scRNA-seq) of RA synovium, we identified a *PLAUR*/uPAR-high myeloid subset that co-expresses pathogenic mediators, including *IL1B* and *CXCL8*. To target these cells, we developed anti-uPAR antibody–drug conjugates (ADCs) and evaluated various payloads in vitro and in vivo. ADCs bearing BCL-2 family inhibitors selectively induced apoptosis in proinflammatory human monocytes and macrophages with elevated uPAR, while sparing unstimulated monocytes with low basal uPAR in vitro. The treatment also reduced CXCL8 secretion. Given that murine myeloid cells exhibited lower uPAR expression and reduced sensitivity to BCL-2 family inhibitors, we used a monomethyl auristatin F (MMAF) payload to demonstrate in vivo proof-of-concept. In an air-pouch model, the anti-uPAR–MMAF conjugate reduced uPAR^high^CD11b^+^F4/80^+^ macrophages by 39% compared with the isotype control. Together, our study underscores the potential of ADCs to eliminate disease-relevant cell types with inducible cell surface markers. This work opens new avenues for exploring cytotoxic ADCs as targeted therapies for autoimmune and inflammatory diseases.

## 1. Introduction

Rheumatoid arthritis (RA) is a chronic autoimmune disease affecting approximately 0.24% to 1% global population [1]. Innate immunity plays a central role in initiating and driving RA pathogenesis [2]. Among innate immune cells, macrophages are key producers of cytokines, chemokines, and degradative enzymes that drive joint inflammation and ultimately lead to cartilage and bone destruction [3]. Recent advances in single-cell RNA-sequencing (scRNA-seq) have provided unprecedented resolution of myeloid cell heterogeneity within inflamed joints, pinpointing disease-relevant myeloid cell states [4,5,6]. These insights offer new opportunities for early drug discovery by enabling the development of cell-targeting therapeutics aimed at selectively depleting pathogenic myeloid cell subsets, with the potential to achieve better therapeutic responses compared to current treatments that target individual cytokines.

Antibody–drug conjugates (ADCs) represent a promising class of cell-targeting therapeutics that utilize monoclonal antibodies (mAbs) to deliver cytotoxic or modulatory payloads specifically into pathogenic cells via cell surface proteins [7]. Depending on the payload’s mechanism of action, ADCs can either modulate or deplete target cells. While ADCs have achieved significant success in oncology [7,8], their application in immunological diseases remains limited. The most advanced immunology ADC is ABBV-3373, a human anti–TNF mAb conjugated to a glucocorticoid receptor modulator (GRM), which completed an evaluation in healthy volunteers and adults with moderate-to-severe RA [9,10]. Two phase-2 studies of a slightly modified, subcutaneously deliverable TNF-GRM ADC derived from ABBV-3373, for polymyalgia rheumatica (ClinicalTrials.gov identifier: NCT04972968) and Crohn’s disease (ClinicalTrials.gov identifier: NCT05068284), were terminated. Other efforts, such as anti-CD163 dexamethasone conjugates targeting macrophages and anti-CD74 conjugates delivering fluticasone propionate analogs to B cells, have not progressed beyond preclinical stages [11,12,13]. Challenges in developing ADCs for immunological indications include a limited repertoire of suitable payloads and a scarcity of well-characterized cell surface markers that can distinguish pathogenic immune cells from non-pathogenic counterparts, thereby minimizing the risk of broad immunosuppression.

In this study, we leveraged recent scRNA-seq findings from RA patients and identified the urokinase plasminogen activator receptor (uPAR), encoded by *PLAUR*, as a highly expressed cell surface marker on an inflammation-associated *IL1B*^+^ proinflammatory myeloid subset. We explored BCL-2 family protein inhibitors as a novel payload class beyond glucocorticoids to induce non-immunogenic cell apoptosis in primary human monocyte-derived macrophages. To generate proof-of-concept in vivo data, we employed an anti-uPAR mAb conjugated with monomethyl auristatin F (MMAF) to demonstrate selective depletion of uPAR^high^-expressing macrophages in a myeloid-rich rodent model. Our study highlights the potential of ADCs to eliminate disease-relevant cell types and provides a practical framework for translating scRNA-seq discoveries into targeted cell-depleting therapies for autoimmune and inflammatory diseases.

## 2. Materials and Methods

### 2.1. Single-Cell RNA-Seq Analysis of Inflammatory Markers in Myeloid Cell Compartment

The myeloid compartment of the Accelerating Medicines Partnership (AMP) Phase 2 single-cell RNA-sequencing dataset from RA patients was analyzed [5]. We applied a standard Seurat pre-processing workflow. Cells were retained if they met all of the following criteria: >500 detected genes per cell (nFeature), <20% mitochondrial reads, and not predicted to be doublets by Scrublet. After filtering, counts were normalized and variance stabilized using SCTransform, and dimensionality reduction was performed with PCA followed by UMAP. An initial clustering resolution of 0.8 was used to detect clusters, which were then grouped into high-level cell populations (myeloid cells, B cells, T cells, and fibroblasts). To evaluate cluster robustness, we examined several resolutions (0.2–1.0) using the FindClusters function in Seurat, performed a visual inspection of UMAP separability, and confirmed quantitatively that key marker expression persisted across resolutions. For marker expression interrogation, we calculated the mean normalized expression and the percentage of cells expressing each gene.

The myeloid compartment, which was selected for further interrogation in this study, showed enrichment for canonical myeloid markers including *ITGAM*, *ITGAX*, *CD14*, *FCGR3A*, and *CD33*. This compartment was subclustered at a resolution of 0.2, yielding 14 transcriptionally distinct clusters. Clusters annotated as doublets or as non-myeloid contamination were excluded from downstream analyses based on marker expression and cluster composition. To define myeloid subtypes, differential expression analysis was performed for each cluster versus all others using Seurat’s FindAllMarkers function. Default parameters were used except where noted: only.pos = TRUE, min.pct = 0.1, and min.diff.pct = 0.1. Genes meeting statistical and expression thresholds (adjusted *p* value ≤ 0.05) were retained for downstream annotation. The DE genes for each cluster were reviewed together with known myeloid subtype markers to assign cluster identities. The full list of DE genes for each subcluster is available in Supplemental Table S1. Cluster 1, annotated as *IL1B*^+^ inflammatory macrophages, was prioritized for further analysis because it exhibited the highest IL1B expression and elevated expression of proinflammatory chemokines (*CCL3*, *CXCL8*, and *CXCL2*) and growth factors (*HBEGF* and *EREG*). Putative cell-surface proteins among the cluster’s DE genes (which included *PLAUR*) were identified by cross-referencing the retained gene list with Human Protein Atlas annotations; genes annotated as “Receptors” (cell surface or membrane localized) were flagged as candidate surface markers.

### 2.2. Primary Human Inflammatory Monocyte and Macrophage Differentiation

For inflammatory monocyte differentiation, 60,000 human peripheral blood (PB) monocytes (StemCell Technologies; Seattle, WA, USA; 70034) were differentiated in RPMI 1640 medium (Thermo Fisher Scientific; Waltham, MA, USA; A1049101) supplemented with 10% heat-inactivated FBS (Thermo Fisher Scientific; Waltham, MA, USA; A5670801), 50 ng/mL GM-CSF (Biolegend; San Diego, CA, USA; 572903), 20 ng/mL TNFα (Peprotech; Waltham, MA, USA; 300-01A), and 300 nM PGE_2_ (Sigma; St. Louis, MO, USA; P0409), in 1 well of a 96-well plate for 3 days. For macrophage differentiation, 60,000 human PB monocytes were cultured in RPMI 1640 medium supplemented with 10% heat-inactivated FBS and 50 ng/mL GM-CSF in 1 well of a 96-well plate for 6–7 days. Fully differentiated macrophages were further polarized using a cocktail of 20 ng/mL TNFα and 300 nM PGE_2_, or 10 ng/mL LPS (Thermo Fisher Scientific; Waltham, MA, USA; 00-4976-93) and 20 ng/mL IFN-γ (R&D systems; Minneapolis, MN, USA; 285-IF/CF) for another 3 days. For polarizing cells into anti-inflammatory macrophages, monocytes were first differentiated from complete media with 50 ng/mL M-CSF (R&D systems; Minneapolis, MN, USA; 216-MC-010/CF) for 7 days, and fully differentiated macrophages were cultured with 10 ng/mL IL-4 (R&D systems; Minneapolis, MN, USA; 204-IL-010/CF) for 3 days.

### 2.3. THP1 Differentiation

Human THP1 cells were purchased from ATCC and cultured in RPMI 1640 medium (Thermo Fisher Scientific; Seattle, WA, USA; A1049101) supplemented with 10% heat-inactivated FBS and 0.05 mM 2-mercaptoethanol (Gibco^TM^; Seattle, WA, USA; 21985023). 0.1 × 10^6^ THP1 cells were seeded into 1 well of a 96-well plate with 100 nM phorbol 12-myristate 13-acetate (Cayman Chemicals; Ann Arbor, MI, USA; 10008014). Cell cultures were changed to fresh media the next day and replaced with media containing 100 pg/mL LPS + 20 ng/mL IFNy the day after for another day.

### 2.4. Mouse Peritoneal Macrophage In Vitro Stimulation

Frozen mouse peritoneal macrophages isolated from the peritoneal cavity of pathogen-free C57BL/6 mice with thioglycolate elicitation were purchased from CellBiologics (Chicago, IL, USA; C57-6032TF) and cultured in RPMI 1640 medium supplemented with 10% heat-inactivated FBS and 50 ng/mL mouse GM-CSF (R&D systems; Minneapolis, MN, USA; 415-ML-010/CF), 50 ng/mL mouse M-CSF (R&D systems; Minneapolis, MN, USA; 416-ML-010/CF), or 20 ng/mL mouse TNFα (R&D systems; Minneapolis, MN, USA; 410-MT-025/CF), as described in the results, for 3 days.

### 2.5. Flow Cytometry and uPAR Cell Surface Copy Number Numeration

Isolated CD14^+^ peripheral blood monocytes or frozen mouse peritoneal macrophages were seeded at 2 × 10^6^ per well in an ultra-low attachment 6-well plate (Corning; Corning, NY, USA) and differentiated into inflammatory monocytes or macrophages as described above. Cells were detached by Detachin™ Cell Detachment Solution (AMSbio; Cambridge, MA, USA; AMS.T100100) within 6 min to avoid cell surface uPAR shedding. A total of 1 × 10^5^ cells were resuspended in 100 μL of autoMACS Running Buffer (Miltenyi Biotech; Gaithersburg, MD, USA) for each antibody staining. Five microliters of the human Fc blocking solution (BioLegend; San Diego, CA, USA; 422302) and the live/dead dye (Thermo Fisher Scientific; Waltham, MA, USA; L10119) were added prior to antibody staining over ice according to the manuals. Human myeloid cells were stained with anti-uPAR mAb (Biolegend; San Diego, CA, USA; 336906), and mouse peritoneal macrophages were stained with anti-mouse uPAR mAb (R&D systems; Minneapolis, MN, USA; FAB531P-100UG), CD11b mAb (Biolegend; San Diego, CA, USA; 101236), and F4/80 mAb (Biolegend; San Diego, CA, USA; 123108) over ice for 45 min. Cells were then washed twice with autoMACS Running Buffer and subject to flow analysis by using FACSymphony™ A3 (BD; Franklin Lakes, NJ, USA). The same parameters were used for running the PE Quantibrite quantification kit (BD; Franklin Lakes, NJ, USA; 340495). Cells were first gated based on forward (FSC-A) and side (SSC-A) scatters to exclude debris. Single cells were then selected based on FSC-A versus FSC-W parameters. Dead cells were excluded based on the positive staining of the Live/Dead dye. The positive cell-surface staining of uPAR of gated live cells was determined by comparing them to fluorescence-minus-one negative controls. Mean fluorescence intensity was used to calculate cell surface copy numbers based on the manual.

### 2.6. Antibody Generation

Recombinant antibodies were expressed using the ExpiCHO™ Expression System (Thermo Fisher Scientific; Waltham, MA, USA) following the manufacturer’s standard protocol. ExpiCHO-S™ cells were cultured in ExpiCHO™ Expression Medium and seeded at 6 × 10^6^ viable cells/mL for transfection. Plasmid DNA was complexed with ExpiFectamine™ CHO Reagent (Thermo Fisher Scientific; Waltham, MA, USA; A29130) in OptiPRO™ SFM and added to the cells. ExpiCHO™ Enhancer and ExpiFectamine™ CHO Transfection Feed were supplied to the culture 18–22 h post-transfection. The culture was maintained at 37 °C with 8% CO_2_ on an orbital shaker.

Anti-mouse uPAR antibodies were generated by immunizing SD rats with mRNA, recombinant murine uPAR-Fc fusion, or murine uPAR-expressing HEK293F cells. Candidate antibodies were selected from FACS cell binding and internalization assessments with murine uPAR-expressing CHO-K1 cells. Domain mapping and epitope binning were evaluated via ELISA with recombinant Fc fusions of FL uPAR or single domain 1, 2, or 3. Antibodies that were functionally similar to 2G10—do not block binding to uPA and internalize upon binding to uPAR—were prioritized for ADC generation to evaluate depletion of uPAR-expressing cells in vitro and in vivo.

### 2.7. Purification and Characterization

On day 6 post-transfection, the cell culture was harvested by centrifugation at 4000 rpm for 20 min. The resulting supernatant was clarified and subjected to affinity purification using a gravity-flow column packed with MabSelectPrismA resin (Cytiva; Marlborough, MA, USA; 10336984). After loading, the resin was washed, and the bound antibody was eluted using Pierce™ IgG Elution Buffer (Thermo Fisher Scientific; Waltham, MA, USA; 21004). The pH of the elution fractions was immediately neutralized with 1 M Tris-HCl, pH 9.0 (Teknova; Hollister, CA, USA; T1090). Subsequently, the sample was buffer-exchanged into phosphate-buffered saline (PBS) at 7.4 pH. The purity of the final antibody preparation was assessed by analytical size-exclusion chromatography (SEC), confirming a monomeric purity of >95% before being transferred for conjugation.

### 2.8. Generation of Anti-uPAR-BCL Conjugates

Four milligrams of anti-uPAR mAb, 2G10, or isotype control mAb was added to a tube with 6.4 uL (2.5 equivalents) of a 10 mM TCEP solution (Millipore Sigma; St. Louis, MO, USA; CAS#: 51805-45-9) in water, and the solution was mixed at 37 °C for 2 h. Then, 10% DMSO was added, and the reaction was cooled to RT and split into two tubes. 7.5 uL (6 equivalents) of the ABT-263-linker-playload or A-1331852-linker-payload at 10 mM stock solutions in DMSO was added to the tubes containing mAbs, and the reactions were shaken at 450 rpm for 3 h. The ADCs were purified via the HiTrap Desalting column (Cytiva; Marlborough, MA, USA; 29048684)/AKTA eluting with PBS, and their concentrations were measured by Nanodrop. The drug-to-antibody ratio (DAR) was determined by mass spectrometry. The average DAR for isotype control-ADCs is 2, and the average DAR for 2G10-ADCs is 3.

### 2.9. Generation of Anti-uPAR-MMAF Conjugates

Nine milligrams of the isotype control or anti-uPAR mAb, mAb028, were added to a tube with 37 uL (6 equivalents) of a 10 mM TCEP solution (Millipore Sigma; St. Louis, MO, USA; CAS#: 51805-45-9) in water. The solution was heated to 37 °C for 3 h and then cooled to RT. 10% DMSO was added, followed by 30 uL (5 equivalents) of a 10 mM DMSO solution of Mc-Val-Cit-PAB-MMAF linker-payload. The reactions were shaken at 450 rpm for 2 h and then purified via the HiTrap desalting column (Cytiva; Marlborough, MA, USA; 29048684)/AKTA eluting with PBS, and their concentrations were measured by nanodrop. The DAR was determined by mass spectrometry. The average DAR for isotype control-ADCs or mAb-028-MMAF conjugate is 3.

### 2.10. Live-Cell Imaging and Analysis

To assess antibody internalization using the pHrodo dye, anti-uPAR or isotype control antibodies were conjugated with the pHrodo deep red dye using the labeling kit (Thermo Fisher Scientific; Waltham, MA, USA; P35355) according to the manual. Human monocytes or THP1 cells were differentiated in a 96-well clear-bottom black microplate (Corning; Corning, NY, USA; 3603) as described previously. Conjugated antibodies with different concentrations indicated in the results were added to cells one day post-LPS and IFN-γ stimulation. Cells were imaged by both phase contrast and red fluorescence channels using the IncuCyte S3 instrument (Sartorius; Bohemia, NY, USA) at 10× magnification every 2 h for 48 h. Cells were maintained at 37 °C and 5% CO_2_ in an incubator. All images were processed using the IncuCyte S3 basic analyzer, which quantified the red channel integrated intensity and phase area per image. Data were shown as integrated intensity normalized to phase area.

To assess antibody trafficking by confocal imaging, 2G10 or isotype control antibodies were conjugated with the ReadyLabel^TM^ AlexaFluor^TM^ 488 Antibody Labeling kit (Thermo Fisher Scientific; Waltham, MA, USA; R10706) according to the manual. Human monocytes were differentiated in a 96-well glass-bottom black plate (CellVis; Mountain View, CA, USA; P96-1.5H-N) as described previously in GM-CSF-containing media for 7 days. Cells were then polarized with TNFa and PGE_2_ combo for another day. On the day of imaging, cells were treated with SiR-lysosome labeling dye (SPIROCHROME, CY-SC012, Stein-am-Rhein, Switzerland) at a final concentration of 10 nM for 3 h at 37 °C and 5% CO_2_ in an incubator. The nuclear Hoechst dye 33342 (Thermo Fisher Scientific; Waltham, MA, USA; 62249) at a final concentration of 1 µg/mL was added to the cell culture for another 10 min. Cell plates were then washed twice with PBS, and then 150 μL of TNFα, PGE_2_, and 10 nM SiR-lysosome labeling dye-containing media was added, with either 67 nM conjugated 2G10 or isotype control mAbs. Cells were immediately subjected to confocal imaging with a 63× objective in Opera Phenix (Revvity; Waltham, MA, USA; HH2400) for 16 h at 37 °C and 5% CO_2_. Imaging channels included AlexaFluor 488 (mAbs), AlexaFluor 647 (SiR-lysosome), and DAPI (Hoechst). Signals Image Artist (Revvity; Waltham, MA, USA) was used for image analytics. The signals of mAbs in lysosomes were measured based on lysosomes segmentation by SiR-Lysosome; Pearson correlation of AF488 (mAbs) and AF647 (SiR-lysosome) was quantified within the cellular region, segmented by cellular autofluorescence.

### 2.11. ADC Treatment and XTT Assay

To test the potency of ADCs in killing inflammatory monocytes, a total of 60,000 primary monocytes in 100 μL of complete RPMI-1640 media supplemented with 50 ng/mL GM-CSF, 20 ng/mL TNFα, and 300 nM PGE_2_ were added to 1 well of a TC-treated 96-well plate. Anti-uPAR mAbs, inhibitors, controls, or anti-uPAR conjugates at indicated concentrations were added to cell cultures to bring the final volume of media in each well to 150 μL. For monocyte killing, 60,000 primary monocytes were used in 100 μL of complete RPMI-1640 media supplemented with 20 ng/mL TNFα. The cells were then treated with different reagents, as described above. The cells were maintained at 37 °C and 5% CO_2_.

To test the killing of ADCs in proinflammatory macrophages, a total of 60,000 primary monocytes in 100 μL of complete RPMI-1640 media supplemented with 50 ng/mL GM-CSF were added to 1 well of a TC-treated 96-well plate for 4 days. Then, 50 μL of differentiation media containing 50 ng/mL GM-CSF was added to each well for another 3 days. Seven days after macrophage differentiation, cells were cultured in fresh polarization media containing 20 ng/mL TNFα and 300 nM PGE_2_ and then treated with anti-uPAR mAbs, inhibitors, controls, or anti-uPAR conjugates at indicated concentrations to generate dose–response curves. The final volume of media in each well was 150 μL. The cells were maintained at 37 °C and 5% CO_2_.

To test the killing of ADCs in mouse peritoneal macrophages, a total of 50,000 frozen mouse peritoneal macrophages (CellBiologics; Chicago, IL, USA; C57-6032TF) in 100 μL of complete RPMI-1640 media supplemented with 50 ng/mL GM-CSF were added to 1 well of a TC-treated 96-well plate. After 3 days, anti-mouse uPAR conjugates, isotype control conjugates, or inhibitors at different concentrations were added to the cell culture to bring the final volume of media in each well to 150 μL. The cells were maintained at 37 °C and 5% CO_2_.

After 3 days of treatment, cell viability was determined using the XTT kit (Cell Signaling; Danvers, MA, USA; 9095) according to the manufacturer’s manual. In short, 50 μL of XTT detection solution was added to each well of the 96-well plate containing cell cultures. The plates were incubated at 37 °C and 5% CO_2_ for another 1 to 2 h and then read using a Spectra Max M5 (Molecular Devices; San Jose, CA, USA). The percentage of cell viability was normalized to the average values of untreated controls.

### 2.12. Annexin V/PI Staining

To assess the types of cell death induced by ADCs, human primary monocytes were differentiated into proinflammatory macrophages in an ultra-low attachment 24-well plate (Corning), as described above. Cells were treated with anti-uPAR conjugates at different concentrations for 3 days. Cells were detached by Detachin™ Cell Detachment Solution (AMSbio; Cambridge, MA, USA; AMS.T100100) and then stained with annexin V and PI dyes from the dead cell apoptosis kit (Thermo Fisher Scientific, V13242) according to the manual. Cells were then detected by FACSymphony™ A3 (BD; Franklin Lakes, NJ, USA).

### 2.13. CXCL8 Quantification Assay

Human monocytes were differentiated into macrophages for 7 days in 96-well plates and polarized into proinflammatory macrophages together with ADC treatment, as described above. After 3 days of treatment, the supernatant was carefully removed from each well. The plates were washed 3 times with PBS, and cells were restimulated with 50 ng/mL GM-CSF-containing media for another 2 days. Supernatant was collected from each well, analyzed using the V-PLEX Proinflammatory Panel 1 Human Kit (Meso Scale Discovery; Rockville, MD, USA) according to the manual, and measured by a SECTOR Imager S 600 (Meso Scale Discovery). Calibrator blends provided in kits were added and analyzed together with samples in the same plates. DISCOVERY WORKBENCH software version 1 (Meso Scale Discovery; Rockville, MD, USA) was used to calculate sample concentrations.

### 2.14. Fab-ZAP Antibody Internalization Assay

Mouse-uPAR-overexpressing CHO-K1 cells (ChemPartner; Watertown, MA, USA) were cultured in Ham’s F-12K medium supplemented with 10% heat-inactivated FBS. The Fab-ZAP rat kit (Advanced Targeting systems; Carlsbad, CA, USA; IT-55) was used to test the internalization capacity of anti-mouse uPAR mAbs. On day 1, a total of 0.5 × 10^6^ cells in 90 μL of media was added to 1 well of a 96-well plate (Corning, 3903). On day 2, the cell plate was treated with different concentrations of anti-mouse uPAR mAbs, Saporin, the positive control, isotype-IgG ZAP, and the negative control according to the manual of the Fab-ZAP kit. On day 5, the cell viability was measured by using the CellTiter-Glo kit (Promega; Madison, WI, USA; G7571), and data were acquired by a PHERAstar plate reader (BMG Labtech; Cary, NC, USA). The percentage of cell viability was normalized to the average values of untreated controls. The mAbs that can induce uPAR-mediated internalization will show a dose-dependent killing curve.

### 2.15. Animal Model and Immunophenotyping

Female C57BL/6J mice were purchased from Jackson Laboratories. All procedures were approved by the Merck & Co., Inc., Rahway, NJ, USA Institutional Animal Care and Use Committee (IACUC). Mice were housed under standard conditions with ad libitum access to food and water.

The mouse air pouch model was used for all the studies. A subcutaneous air pouch was created by injecting 5 mL of sterile air into the back of a mouse on day 1. To quantify cell surface uPAR density, on day 5, the air pouch was reinflated by 2 mL of sterile air to induce an inflammatory response. Mice were sacrificed on day 7, and cells were collected from the exudate for downstream analysis. For MMAF conjugate treatment, mice were i.p injected with 10 mg/kg anti-uPAR-MMAF, isotype control-MMAF, or PBS control on day 4. On day 5, the air pouch was reinflated by 2 mL of sterile air again to induce inflammation. Mice were sacrificed on day 7, and blood was collected. The air pouch was flushed with 1 mL cold PBS, massaged, and the exudate was collected at the same time point.

Cells collected from exudate were resuspended in the autoMACS Running Buffer (Miltenyi Biotech; Gaithersburg, MD, USA) containing the Brilliant Stain Buffer (Thermo Fisher Scientific; Waltham, MA, USA; 00-4409-42), the live/dead dye (Thermo Fisher Scientific; Waltham, MA, USA;L10119), and Fc blockers (Biolegend; San Diego, CA, USA; 101320) according to the manual. Cells were then stained with the following immunophenotyping flow mAbs: Ly6C (BD; Franklin Lakes, NJ, USA; 553104), Ly6G (BD, 562737), F4/80 (Biolegend, 123141), CD45 (BD, 564279), CD11b (BD, 564443), CD3 (BD, 561108), CD19 (BD, 551001), and CD49b (Biolegend, 108916). To assess cell surface uPAR density, anti-mouse uPAR mAb (R&D, FAB531P-100UG) was used to stain uPAR. The PE Quantibrite quantification kit (BD, 340495) was used for quantification. To determine cell surface uPAR occupancy by mAb028-MMAF, mAb019 conjugated with Alexa Fluor 647 by the antibody labeling kit (Thermo Fisher Scientific; Waltham, MA, USA; A88068) was used to stain cell-surface uPAR. Cells were incubated with flow antibodies over ice for 40 min and then subjected to flow analysis. To count cell number, CountBright™ Absolute Counting Beads (Thermo Fisher Scientific; Waltham, MA, USA; C36950) were used. Flow data acquisition and analysis were performed as previously described in the Flow Cytometry method section.

### 2.16. Plasma ADC Concentration Measurement

ADC concentrations were determined using a conjugated payload assay. The conjugated payload associated with the uPAR-MMAF conjugate and an isotype control–MMAF conjugate was measured in mouse plasma by affinity capture, on-cartridge proteolytic release, and LC-MS/MS quantification. Briefly, biotinylated anti-human IgG F(ab’)^2^ (Jackson ImmunoResearch; West Grove, PA, USA; 109-066-088) was loaded onto 5 µL streptavidin cartridges (Agilent; Santa Clara, CA, USA; G5496-60010) using an AssayMap Bravo (Agilent; Santa Clara, CA, USA). Diluted plasma samples and ADC standards in blank mouse plasma were applied to the cartridges, which were washed on the platform to remove unbound matrix. The conjugated payload was released by on-cartridge digestion with freshly activated papain (2 mg/mL in 100 mM Tris-HCl, pH 8.0, 2 mM cysteine; Roche, Indianapolis, IN, USA; 10108014001), incubated at 37 °C for 15 min, and eluates were collected.

Eluates were mixed 1:4 (*v*/*v*) with acetonitrile containing 0.1% formic acid and imipramine as internal standard, vortexed, and centrifuged at 4200× *g* for 10 min. Supernatants were analyzed on a Waters ACQUITY UPLC HSS T3 column (1.8 µm, 2.1 × 50 mm) at 40 °C with mobile phases A (0.1% formic acid in water) and B (0.1% formic acid in acetonitrile). The flow rate was 750 µL/min with the following gradient: 95% A/5% B (initial, hold to 0.25 min), linear to 5% A/95% B at 1.75 min (hold to 2.16 min), return to initial at 2.17 min, and re-equilibration to 3.00 min total run time.

Detection was performed by AB SCIEX API 6500 LC-MS/MS (Framingham, MA, USA) with TurboIonSpray using positive-mode MRM; data were acquired with Analyst 1.7.2 and quantified in MultiQuant 3.0.31721 against calibration standards prepared in mouse plasma using linear 1/x^2^ weighting.

### 2.17. Statistical Analysis

Data are shown as mean ± SEM. Statistical tests are indicated in the corresponding figure legends. Differences were considered statistically significant when *p* ≤ 0.05.

## 3. Results

### 3.1. PLAUR/uPAR Expression Is Elevated in the IL1B^+^ Inflammatory Myeloid Cluster Identified in RA Single-Cell RNA-Seq Dataset as Well as In Vitro Differentiated Proinflammatory Monocytes and Macrophages

**Figure 1:**
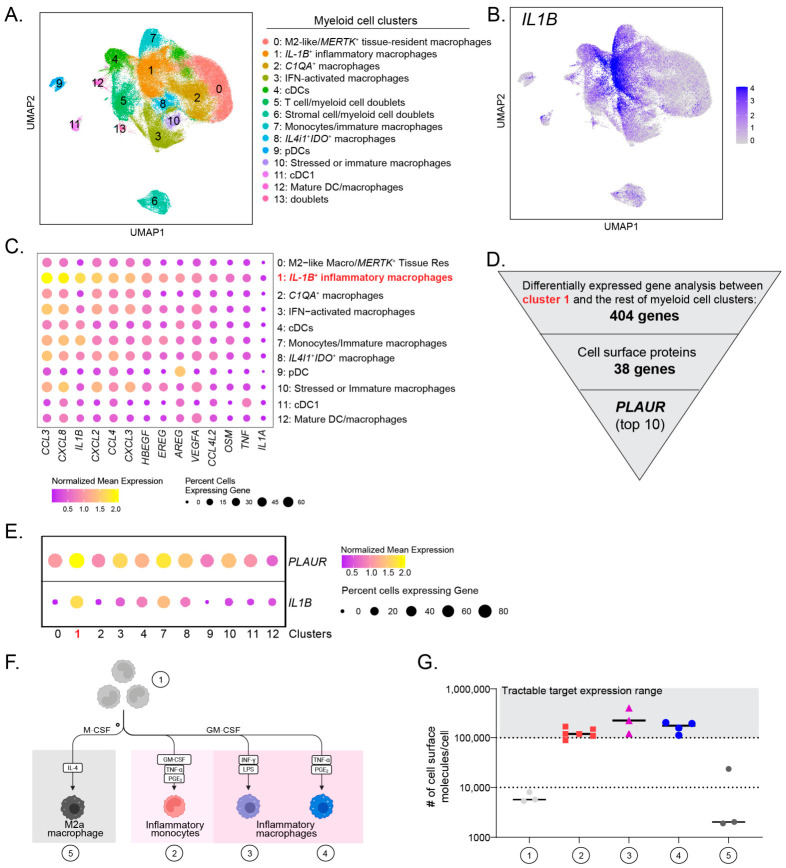
The uPAR expression of myeloid cells at mRNA and protein levels. (**A**) UMAP of myeloid cells (**left**) and annotation of cell clusters (**right**). (**B**) The UMAP color-coded by the relative expression of *IL1B*. (**C**) The dot plot of the mean expression of genes encoding inflammatory and growth factors. (**D**) Workflow of identifying *PLAUR*, as the cell surface marker of cluster 1. (**E**) The dot plot of the mean expression of *PLAUR* and *IL1B* across myeloid cell clusters. (**F**) Schematic illustration of different monocyte to macrophage differentiation conditions; “1” represents human primary monocytes; “2” represents in-vitro-differentiated inflammatory monocytes; “3” represents IFN-γ/LPS-stimulated inflammatory macrophages; “4” represents TNFα/PGE_2_-stimulated inflammatory macrophages; “5” represents M2a macrophages. (**G**) The quantification of cell surface uPAR copy number (#) in different myeloid cell subsets. “1” represents human primary monocytes; “2” represents in-vitro-differentiated inflammatory monocytes; “3” represents IFN-γ/LPS-stimulated inflammatory macrophages; “4” represents TNFα/PGE_2_-stimulated inflammatory macrophages; “5” represents M2a macrophages.

To identify a myeloid cell cluster of interest for therapeutic targeting, we re-analyzed the myeloid compartment in the Accelerating Medicines Partnership (AMP) phase 2 single-cell RNA-seq dataset derived from RA patients [5]. We identified 14 distinct clusters, with clusters 5, 6, and 13 classified as doublets or contamination of other non-myeloid cell types (Figure 1A). Among these, cluster 1 has the highest expression of *IL1B* (Figure 1B). Besides *IL1B*, detailed analysis revealed that cluster 1 also expressed elevated levels of pro-inflammatory chemokines (*CCL3*, *CXCL8*, and *CXCL2*), growth factors (*HBEGF*, *EREG*), and the pro-fibrotic factor *OSM* (Figure 1C). This cluster likely corresponds to the previously described “M-7” subset characterized by high *IL1B* and *HBEGF* expression in the study by Zhang et al. [5]. Notably, *HBEGF*^+^ inflammatory macrophages have also been identified in an independent RA scRNA-seq dataset [6]. Given the expansion of the *IL1B*^+^*HBEGF*^+^ myeloid cluster in RA patients and its reported critical role in RA pathogenesis [4,6], we sought to identify a cell surface marker that could specifically capture this inflammatory myeloid subset. We performed differential expression (DE) analysis, comparing cluster 1 to other myeloid clusters and filtered for genes encoding cell surface proteins (Figure 1D). From 38 candidate genes, we ranked them based on fold change and the percentage of cluster 1 cells expressing each gene (Supplementary Table S1). *PLAUR* (the gene encoding uPAR) emerged as the top candidate, being highly expressed in over 95% of cells within cluster 1 (Figure 1E, Supplementary Table S2).

To validate the cell surface expression of uPAR, we differentiated primary human CD14^+^ monocytes into classic inflammatory (“M1-like”) or anti-inflammatory (“M2-like”) macrophages in vitro (Figure 1F). Previous studies have shown that treatment with TNFα combined with synovial fibroblast-derived prostaglandin E2 (PGE_2_) upregulates *PLAUR* expression and recapitulates the gene signature of *IL1B*^+^*HBEGF*^+^ macrophages identified in an RA scRNA-seq dataset [6]. Accordingly, we included this treatment to model disease-associated *IL1B*^+^*PLAUR*^high^ inflammatory monocytes, an intermediate cell state in monocyte-to-macrophage differentiation, as well as fully differentiated macrophages. Measurement of cell surface uPAR density revealed that pro-inflammatory monocytes and macrophages exhibited copy numbers exceeding 100,000, a tractable target range for ADCs [14], whereas unstimulated monocytes and M2-like macrophages showed levels around or below 10,000 (Figure 1G).

Together, these data indicate that although PLAUR/uPAR expression is highly enriched in disease-associated IL1B^+^ inflammatory myeloid cells, it is not exclusive to these cells.

### 3.2. Identification of an Anti-uPAR mAb 2G10 That Induces uPAR-Mediated Internalization

**Figure 2:**
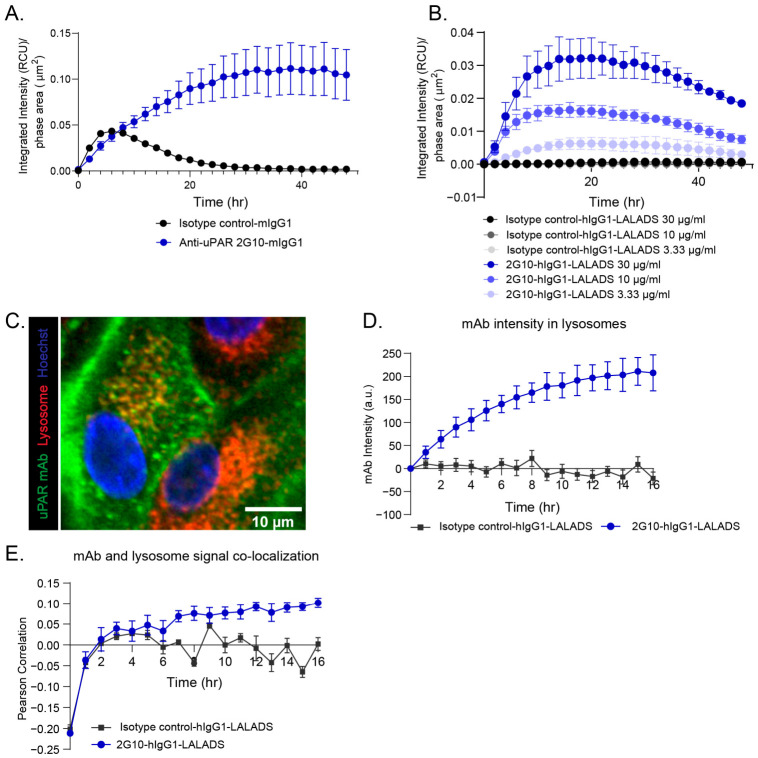
Internalization of anti-uPAR mAb 2G10 in myeloid cells. (**A**) The integrated fluorescence intensity of pHrodo dye-conjugated isotype control and 2G10 mAbs in monocyte-derived M1 macrophages at different time points. (**B**) The integrated fluorescence intensity of pHrodo dye-conjugated isotype control and 2G10 mAbs at indicated concentrations in LPS/IFNγ-stimulated THP1 cells at different time points. The increased fluorescence intensity of 2G10 mAbs indicates uPAR-mediated internalization. (**C**) A representative image of anti-uPAR mAb internalization at 3 h after mAb treatment; 67 nM of mAbs labeled with AlexaFluor488 (green color) was used for imaging. Hoechst dyes (blue color) were used for staining cell nucleus, and SiR-Lysosome (red color) was used for staining lysosomes. Scale bar: 20 µm. (**D**) Quantification of the intensities of isotype control or 2G10 mAb in lysosomes. (**E**) The Pearson correlation of the co-localization of mAb and lysosome signals.

A previous study demonstrated that the anti-uPAR mAb clone 2G10 can internalize upon binding to uPAR expressed on breast cancer cell lines [15]. We sought to evaluate whether 2G10 could similarly induce internalization of in vitro-differentiated human proinflammatory myeloid cells. To this end, we conjugated 2G10 and its isotype control with the pH-Rodo dye, which fluoresces in acidic organelles such as late endosomes and lysosomes—key compartments where ADCs traffic to release their payloads [14]. Human primary monocytes differentiated into inflammatory macrophages polarized with IFNγ/LPS were treated with pH-Rodo dye-conjugated 2G10 or isotype control. We observed a significant increase in fluorescence intensity in the 2G10-treated group compared to the isotype control, indicating that 2G10 induces uPAR-mediated internalization in inflammatory macrophages (Figure 2A).

Since myeloid cells, particularly macrophages, can internalize ADCs via Fcγ receptor-mediated non-specific endocytosis [16], the 2G10 antibody and its isotype control were expressed on a human IgG1 backbone engineered with the Fc-silencing mutations L234A, L235A, and D265S (LALADS) [17] (Supplementary Figure S1A). These mutations abolish Fc-mediated uptake, allowing us to specifically investigate uPAR-mediated internalization rather than non-specific Fc-driven uptake. When IFNγ/LPS-stimulated THP-1 cells were treated with pH-Rodo dye-conjugated 2G10 or the isotype control bearing the LALADS Fc mutation, we observed a dose-dependent increase in fluorescence intensity in the 2G10 group, whereas the isotype control showed minimal fluorescence signal (Figure 2B). This result indicates that Fc engineering with the LALADS mutation does not impair the target-mediated internalization capacity of 2G10.

To further verify trafficking to lysosomes, the desired cell compartment for payload release [18], we performed confocal imaging in monocyte-derived proinflammatory macrophages stimulated with TNFα and PGE_2_. We observed colocalization between anti-uPAR mAb, 2G10, and lysosomal trackers (Figure 2C), with 2G10 intensity in lysosomes increasing over time compared with the isotype control (Figure 2D). Pearson correlation coefficients between 2G10 and lysosome signals also increased over time, while the isotype control showed no positive correlation (Figure 2E). Together, these data demonstrate that 2G10 is internalized via binding to cell surface uPAR and traffics to lysosomes, validating its suitability as a tool antibody for anti-uPAR ADC generation.

### 3.3. Anti-uPAR Drug Conjugates Selectively Deplete uPAR-High-Expressing Human Inflammatory Monocytes and Macrophages

**Figure 3:**
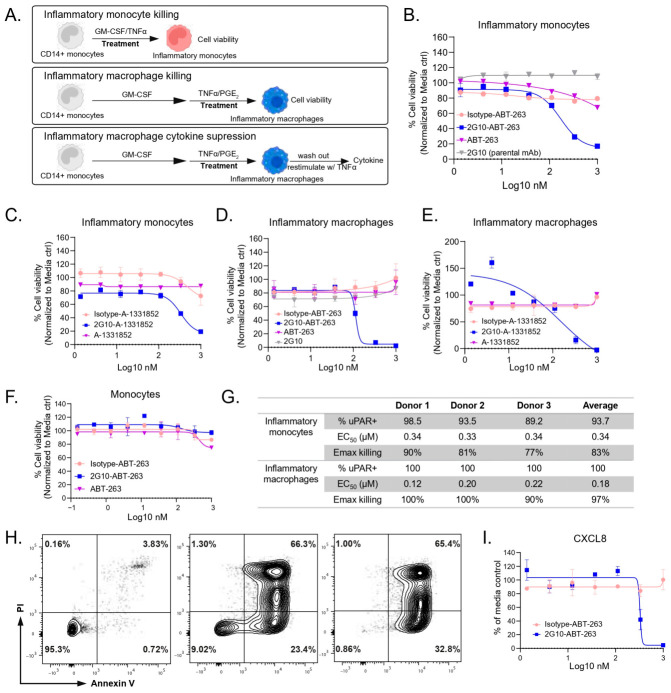
The potency of ADCs in killing different human monocyte-derived myeloid cell subsets in vitro. (**A**) A schematic illustration of ADC treatment. (**B**,**C**) The cell viability of inflammatory monocytes treated with isotype-control, anti-uPAR 2G10 ADCs, parental mAb 2G10, or free payloads (ABT-263 or A-1331852) for 3 days. (**D**,**E**) The cell viability of inflammatory macrophages treated with isotype-control, anti-uPAR 2G10 ADCs, parental mAb 2G10, or free payloads (ABT-263 or A-1331852) for 3 days. (**F**) The cell viability of monocytes treated with isotype-control, anti-uPAR 2G10 ADCs, or the free payloads (ABT-263) for 3 days. (**G**) The summary of percentages of uPAR^+^ cells, EC_50_, and E_max_ killing of the 2G10-ABT-263 ADC across 3 PBMC donors. (**H**) The PI and Annexin V staining result of inflammatory macrophages treated with PBS, 0.3 µM, or 1 µM of 2G10-ABT-263 ADC for 3 days, indicative of an apoptotic cell death. (**I**) CXCL8 concentration of inflammatory macrophages restimulated with TNFα after ADC treatment.

Apoptosis, a tightly regulated form of cell death, plays a crucial role in maintaining myeloid cell homeostasis and preventing disorders like inflammation [19]. Here, we sought to harness the apoptotic pathway to selectively eliminate inflammatory myeloid cell subsets by deploying reported BCL-2 family inhibitors, which antagonize pro-survival proteins and thereby trigger apoptotic cell death in target cells [20]. The anti-uPAR antibody 2G10 and its isotype control were conjugated to either a pan-BCL-2 family inhibitor (ABT-263) [20] or a selective BCL-xL inhibitor (A-1331852) [21] (Supplementary Figure S1B,C). Inflammatory monocytes and macrophages were treated for three days with isotype control conjugates, 2G10 conjugates, free ABT-263, free A-1331852, or parental 2G10 mAb (Figure 3A). The uPAR-targeting conjugates (2G10-ABT-263 and 2G10-A-1331852) efficiently induced cell death in both inflammatory monocytes and macrophages, whereas the other treatment groups had minimal effects on cell viability at the tested concentrations (Figure 3B–E). To assess selectivity, we treated monocytes with low uPAR expression and observed no significant difference in viability between cells treated with anti-uPAR conjugates and isotype control conjugates (Figure 3F), indicating that the uPAR conjugates selectively kill uPAR^high^-expressing cells. Since the ABT-263 conjugates exhibited slightly greater potency than the A-1331852 conjugates, we focused on ABT-263 conjugates for subsequent experiments. This conjugate demonstrated consistent EC_50_ and E_max_ killing values across three donors (Figure 3G).

To characterize the types of cell death, inflammatory macrophages were treated with high doses of 2G10-ABT-263 conjugates (0.3 and 1 µM) and stained with Annexin V and propidium iodide (PI). Over 90% of treated cells exhibited a PI^−^ Annexin V^+^ apoptotic phenotype (Figure 3H), with minimal necrotic cells (PI^+^ Annexin V^−^) even at the highest dose. Furthermore, we evaluated whether the reduction in inflammatory macrophage numbers following uPAR conjugate treatment would decrease cytokine production (Figure 3A). Upon TNFα re-stimulation, CXCL8 production was significantly reduced in the 2G10-ABT-263-treated group (Figure 3I). Collectively, these data indicate that the anti-uPAR BCL-2 inhibitor conjugate can selectively deplete proinflammatory myeloid cells with high uPAR expression and suppress cytokine production.

### 3.4. In Vitro Killing of Mouse Peritoneal uPAR^+^CD11b^+^F4/80^+^ Macrophages by an Anti-Mouse uPAR–MMAF Conjugate

**Figure 4:**
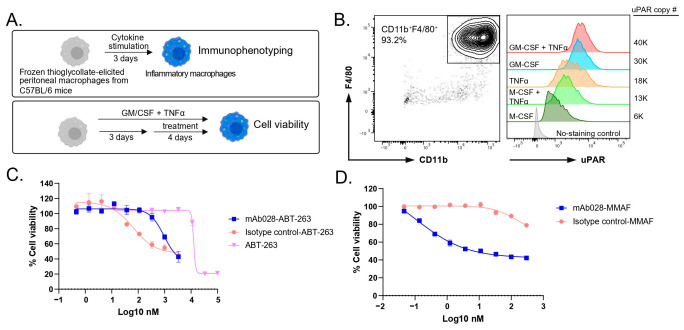
The potency of ADCs in killing proinflammatory mouse peritoneal macrophages in vitro. (**A**) A schematic illustration of proinflammatory macrophage differentiation and ADC treatment. (**B**) Flow cytometry data to show uPAR expression and uPAR copy number (#) of CD11b^+^F4/80^+^ peritoneal macrophages stimulated with different cytokines. (**C**) The cell viability of proinflammatory macrophages stimulated with GM-CSF and TNFα treated with isotype-control, anti-uPAR mAb028 ADCs, or the free payloads (ABT-263) for 4 days. (**D**) The cell viability of proinflammatory macrophages stimulated with GM-CSF and TNFα treated with isotype-control or anti-uPAR mAb028-MMAF conjugates for 4 days.

Next, we sought to demonstrate the selective killing of uPAR-expressing macrophages in the murine system. CD11b^+^F4/80^+^ macrophages were isolated from thioglycolate-elicited peritoneal cavities and stimulated with various cytokines, resulting in detectable cell surface uPAR expression (Figure 4A,B). Since the anti-human uPAR antibody 2G10 does not cross-react with mouse uPAR [15], we generated an anti-mouse uPAR mAb, mAb-028, which binds cell surface uPAR and induces target-mediated internalization (Supplementary Figure S2A). We conjugated mAb-028 and an isotype control antibody with the BCL-2 family inhibitor, ABT-263, and treated peritoneal macrophages with these conjugates. Unexpectedly, the isotype control conjugate exhibited more potent killing than the mAb-028 conjugate, indicating a lack of target-mediated cytotoxicity (Figure 4C). This result was confirmed using a different anti-mouse uPAR clone, mAb-006, which can also induce uPAR-mediated internalization (Supplementary Figure S2A,B), suggesting that the non-specific killing was not due to different clones of anti-uPAR mAbs.

We hypothesized that this non-specific killing could be attributed to (1) relatively low uPAR expression on murine macrophages and (2) reduced sensitivity of murine macrophages to ABT-263-mediated apoptosis (Supplementary Figure S2C,D). Because murine macrophages exhibited reduced sensitivity to BCL-2 inhibition, we conjugated the antibodies with MMAF, a potent cytotoxic payload commonly used in oncology ADCs [22] (Supplementary Figure S2E). In addition, MMAF’s low cell permeability minimizes bystander effects, allowing the demonstration of target-mediated killing [22]. Treatment with the mAb-028-MMAF conjugate resulted in significantly greater killing compared to the isotype control conjugate, indicating effective target-mediated depletion of uPAR-expressing macrophages (Figure 4D).

### 3.5. In Vivo Depletion of CD11b^+^F4/80^+^ Macrophages in an Air-Pouch Model

**Figure 5:**
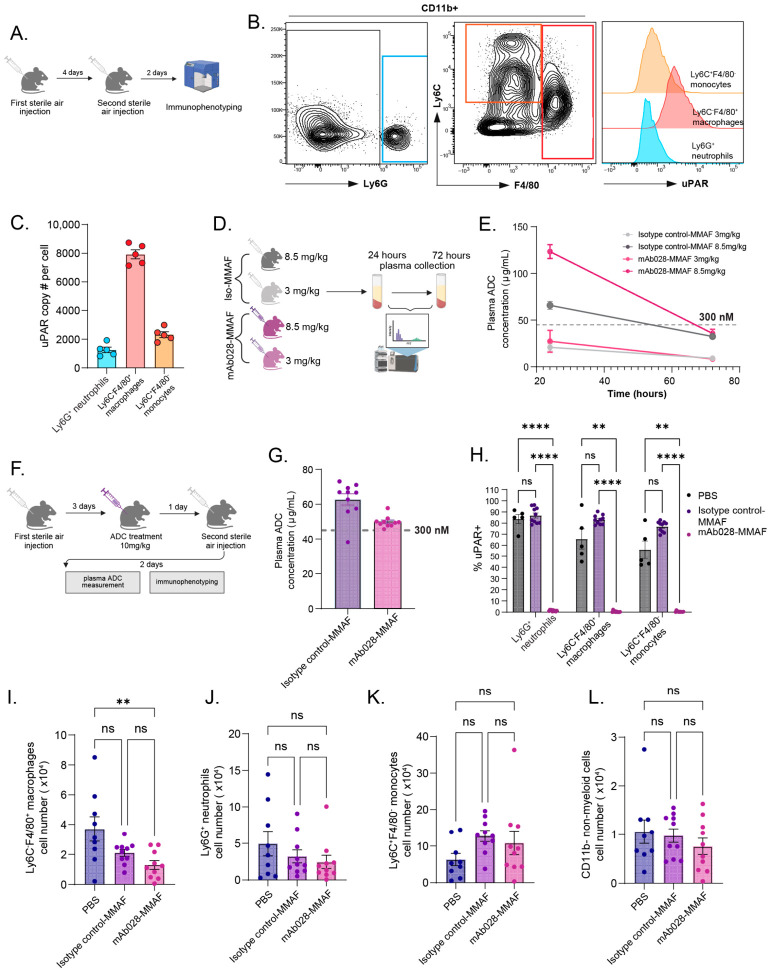
The potency of MMAF ADCs in depleting uPAR^high^-expressing macrophages in vivo. (**A**) A schematic illustration of the air-pouch model used. (**B**) The immunophenotyping of myeloid cells (CD3^−^CD19^−^CD49b^−^CD11b^+^) from exudate. The gated Ly6G^+^ neutrophils (**left panel**), Ly6C^+^F4/80^−^ monocytes and F4/80^+^ macrophages (**middle panel**), and cell surface uPAR expression (**right panel**). (**C**) The quantification of surface uPAR copy number (#) of neutrophils, monocytes, and macrophages (*n* = 5 mice). (**D**) A schematic illustration of ADC dosing for blood collection. (**E**) The plasma concentrations of isotype control- or anti-uPAR MMAF ADC treatment groups at 24 h or 72 h after ADC treatment. (**F**) A schematic illustration of ADC treatment of the air-pouch model. (**G**) The plasma ADC concentrations of animals dosed with 10 mg/kg ADCs at 72 h. (**H**) Quantification of uPAR^+^ cells detected by a competing mAb019 (versus mAb028) across myeloid subsets in PBS, isotype-control–MMAF, and mAb028–MMAF-treated groups. The significantly lower percentage of uPAR^+^ cells detected in the mAb028–MMAF group indicates high surface-uPAR occupancy by the anti-uPAR ADC (mAb028–MMAF). (**I**–**L**) The cell numbers of F4/80^+^ macrophages (**I**), Ly6G^+^ neutrophils (**J**), Ly6C^+^F4/80^−^ monocytes (**K**), and CD11b^−^ non-myeloid cells (**L**) in PBS, isotype-control–MMAF, and mAb028–MMAF-treated groups. n ≥ 5 mice. One-way ANOVA with Turkey’s multiple comparison test was used to calculate statistical significance. **, *p* < 0.01; ****, *p* < 0.0001; ns, not statistically significant.

We next evaluated whether the uPAR-targeting MMAF conjugate could selectively deplete uPAR^+^ cells in vivo using an air-pouch model to assess its ability to target myeloid subsets with varying uPAR expression. The air pouch model is an acute experimental model used to study innate immunity by creating a subcutaneous air-filled cavity in rodents, allowing for characterizing immune infiltrates in the cavity by collecting the exudate [23]. Two days after the second sterile air injection (Figure 5A), we detected CD11b^+^Ly6G^+^ neutrophils, CD11b^+^Ly6C^+^F4/80^−^ monocytes, and CD11b^+^F4/80^+^ macrophages in the air pouch exudate (Figure 5B). All three myeloid subsets expressed uPAR, with F4/80^+^ macrophages exhibiting the highest surface uPAR copy number (~8000 copies per cell) (Figure 5B,C), whereas CD11b- non-myeloid cells had little to no detectable uPAR expression (Supplementary Figure S3A).

Wild-type mice were intraperitoneally (i.p.) injected with either isotype control-MMAF or mAb-028-MMAF at doses of 8.5 mg/kg or 3 mg/kg, respectively. Plasma samples were collected at 24 and 72 h post-injection (Figure 5D), and plasma ADC concentrations were measured (Figure 5E). At 8.5 mg/kg, plasma ADC levels exceeded 300 nM at 24 h but fell below this threshold, the concentration required for maximal in vitro killing (Figure 4D), by 72 h. These data suggest that doses higher than 8.5 mg/kg are necessary to maintain effective plasma ADC concentrations.

Based on this pharmacokinetic data, we i.p. administered 10 mg/kg of MMAF conjugates one day before the second sterile air injection. Plasma ADC concentrations and depletion of cells were assessed two days after the second sterile air injection to induce inflammation (Figure 5F). Both isotype control-MMAF and mAb-028-MMAF were well tolerated with minimal loss of body weight (Supplementary Figure S3B). The 10 mg/kg-dose maintained plasma ADC concentrations above 300 nM in most animals three days post-injection, with only one isotype control-MMAF-treated mouse showing slightly lower levels (Figure 5G). A competing uPAR mAb, mAb-019, to mAb-028 was used to assess target engagement of the antibody arm (Supplementary Figure S2F). uPAR^+^ cells detected by the competing mAb in the mAb028-MMAF group were almost completely reduced compared to the PBS or isotype control-MMAF group in all three myeloid subsets (Figure 5H), indicating efficient target engagement. We then quantified the cell numbers of the three myeloid subsets, as well as CD11b^–^ non-myeloid cells, which do not express uPAR. Compared with PBS-treated animals, uPAR-MMAF produced a significant 65% reduction in CD11b^+^F4/80^+^ macrophages with high uPAR expression (Figure 5I). Relative to the isotype control-MMAF, uPAR-MMAF produced a 39% reduction that did not reach statistical significance (Figure 5I). The activity observed with the isotype control-MMAF suggests non-specific uptake at this dose. The mAb028-MMAF conjugate had minimal impact on uPAR-low monocytes, neutrophils, and non-myeloid cells (Figure 5J–L). Together, these results provide in vivo proof-of-concept that myeloid subsets with high uPAR expression can be selectively depleted.

## 4. Discussion

We leveraged publicly available scRNA-seq datasets to focus on targeting a disease-relevant proinflammatory *PLAUR*^high^*IL1B*^+^ myeloid cell subset identified across multiple RA cohorts [4,5,6]. Zhang and colleagues previously described this subset as *IL1B*^+^ pro-inflammatory monocytes (SC-M1) in the AMP Phase 1 RA scRNA-seq dataset and found that *PLAUR* and *HBEGF* are among the genes associated with *IL1B*^+^ monocytes in bulk RNA-seq monocyte samples from individuals with leukocyte-rich RA and osteoarthritis [4]. Another group similarly identified this myeloid subset characterized by high expression of *HBEGF*, *PLAUR*, and *IL1B*, whose phenotype is shaped by inflammatory cytokines TNFα and PGE_2_ secreted by synovial fibroblasts [6]. This subset contributes to the pathogenic macrophage-fibroblast circuit and promotes synovial fibroblast invasiveness via secretion of epidermal growth factor (EGF) [6]. The authors further demonstrated that FDA-approved medications, including the combination of anti-TNFα and EGFR inhibitors, can disrupt this pathogenic crosstalk by reshaping macrophage and fibroblast phenotypes [6]. Our approach offers a potential strategy to disrupt this pathogenic circuit by selectively depleting macrophages. Together, our work supports the feasibility of targeting uPAR-high myeloid cells and provides evidence for selective depletion strategies, but it does not present a validated therapeutic approach at this stage.

Unlike previous efforts that primarily employed glucocorticoid receptor modulators as payloads [9,11,12,13], our study explores BCL-2 family inhibitors as a novel payload class. Apoptosis is a conserved program essential for immune homeostasis [24]. By exploiting this mechanism, we aim to restore immune balance through the selective induction of apoptosis in inflammatory myeloid cells, thereby avoiding inflammation associated with other forms of cell death. Potent cytotoxic payloads commonly used in oncology ADCs, such as MMAE and topoisomerase I inhibitors, can cause hematological toxicities including pancytopenia [25,26]. However, an anti-B7-H3 monoclonal antibody conjugated with a BCL-xL inhibitor demonstrated a tolerable safety profile without thrombocytopenia or neutropenia in a phase I study of small-cell lung cancer [27]. Our findings align with these clinical observations, showing that BCL-2 family inhibitor conjugates selectively deplete inflammatory monocytes and macrophages with high uPAR expression in vitro, while sparing unstimulated monocytes. This suggests that BCL-2 family inhibitors represent a promising cytotoxic payload class with improved safety profiles suitable for non-oncology indications.

Beyond autoimmune diseases, anti-uPAR conjugates can also be explored for aging-associated pathologies [28,29]. Several studies have identified uPAR as a cell surface marker of senescent cells [28,29]. Depletion of uPAR-expressing cells using chimeric antigen receptor T (CAR-T) cells ameliorated liver fibrosis and age-related metabolic dysfunction in mouse models [28,29]. Given the complexity, manufacturing challenges, and high costs associated with CAR-T therapies [30], ADCs may offer a more accessible, off-the-shelf alternative for prophylactic and therapeutic interventions in age-related diseases.

Our study also highlights several challenges specific to developing ADCs outside oncology. First, non-oncology ADCs require a clean target expression profile to minimize off-target toxicity. In our study, uPAR was largely myeloid-lineage-restricted and further induced by inflammatory stimuli, but this inducibility complicates payload selection and therapeutic-window design. Choosing an appropriate payload is therefore crucial to selectively depleting uPAR-high macrophages. A highly potent uPAR-cytotoxic ADC risks eliminating broader populations of uPAR-expressing myeloid cells, which could cause off-target toxicity and increase susceptibility to infection. Second, the higher safety bar for non-oncology indications limits the use of the highly potent cytotoxic payloads common in oncology, forcing the consideration of alternative payload classes with improved tolerability. For BCL-2 family inhibitor conjugates, optimizing potency while maintaining manufacturability will require strategies to increase DAR without compromising ADC stability or biophysical properties, and careful selection of antibodies that internalize efficiently. Third, translating immunology ADCs from in vitro systems to in vivo models—and establishing meaningful in vitro–in vivo efficacy correlations—is particularly difficult. In oncology, identical human tumor cell lines are often used in both in vitro assays and xenograft models, minimizing discrepancies in target expression and cell-intrinsic payload sensitivity [31]. By contrast, we found that murine myeloid cells express substantially lower levels of uPAR than their human counterparts, even after stimulation with proinflammatory cytokines, and are less sensitive to BCL-2 family inhibitors. To obtain in vivo proof-of-concept, we therefore had to employ a more potent payload (MMAF) to achieve target-mediated depletion, which prevented in vivo testing of our preferred BCL-2 inhibitor conjugates. These issues underscore the need for additional de-risking strategies, such as humanized or knock-in models, surrogate payloads, improved ADC design, and rigorous PK/PD bridging studies, to better align preclinical systems with human biology and accelerate immunology ADC development.

## Data Availability

The AMP phase 2 RA single-cell RNA-sequencing dataset is available: https://immunogenomics.io/ampra2/ (accessed on 5 May 2020 through AMP partnership’s early access). All other raw data are available upon request from the corresponding authors.

## Supplementary Materials

The following supporting information can be downloaded at: https://www.mdpi.com/article/10.3390/cells15090803/s1, Figure S1: The profiling of 2G10-hIgG1-LALADS antibodies and chemical structures of ABT-263 linker-payload and A-1331852 linker payload; Figure S2: The profiling of anti-mouse uPAR mAb, payloads, and ADCs in murine macrophages. (A). The percentage of relative cell viability of mouse uPAR-overexpressing CHO-K1 cells treated with Saporin, Isotype control rat IgG, anti-uPAR mAb-028 and mAb-006 for 3 days. The rat IgG serves as a negative control. mAb-028 and mAb-006 showed dose-response killing, indicative of antibody internalization. (B). The cell viability of proinflammatory mouse peritoneal macrophages stimulated with GM-CSF and TNFα treated with isotype-control, anti-uPAR mAb006 ADCs, or the free payloads (ABT-263) for 4 days. (C,D) The dose-response killing curves of ABT-263 and A-1331852 in human inflammatory macrophages stimulated with TNFα and PGE2 (C) or in mouse peritoneal macrophages stimulated with GM-CSF and TNFα (D). (E) The chemical structure of the MMAF linker-payload: Mc-Val-Cit-PAB-MMAF. (F) Geometric mean fluorescence intensity (GeoMFI) of cell surface uPAR staining by mAb029 of mouse peritoneal macrophages treated with different concentrations of mAb-028-MMAF or Isotype Control-MMAF conjugates. Figure S3: uPAR expression in CD11b^−^ non-myeloid cells and mouse body weight after PBS or ADC treatment. Table S1: Myeloid cell compartment subcluster DE genes used to define and characterize sub-cell types; Table S2: top 15 cell surface markers for cluster 1, the IL1B^+^ myeloid cell cluster.

## References

[1] Almutairi K., Nossent J., Preen D., Keen H., Inderjeeth C. (2020). The global prevalence of rheumatoid arthritis: A meta-analysis based on a systematic review. Rheumatol. Int..

[2] Edilova M.I., Akram A., Abdul-Sater A.A. (2021). Innate immunity drives pathogenesis of rheumatoid arthritis. Biomed. J..

[3] Kinne R.W., Brauer R., Stuhlmuller B., Palombo-Kinne E., Burmester G.R. (2000). Macrophages in rheumatoid arthritis. Arthritis Res..

[4] Zhang F., Wei K., Slowikowski K., Fonseka C.Y., Rao D.A., Kelly S., Goodman S.M., Tabechian D., Hughes L.B., Salomon-Escoto K. (2019). Defining inflammatory cell states in rheumatoid arthritis joint synovial tissues by integrating single-cell transcriptomics and mass cytometry. Nat. Immunol..

[5] Zhang F., Jonsson A.H., Nathan A., Millard N., Curtis M., Xiao Q., Gutierrez-Arcelus M., Apruzzese W., Watts G.F.M., Weisenfeld D. (2023). Deconstruction of rheumatoid arthritis synovium defines inflammatory subtypes. Nature.

[6] Kuo D., Ding J., Cohn I.S., Zhang F., Wei K., Rao D.A., Rozo C., Sokhi U.K., Shanaj S., Oliver D.J. (2019). HBEGF^+^ macrophages in rheumatoid arthritis induce fibroblast invasiveness. Sci. Transl. Med..

[7] Colombo R., Tarantino P., Rich J.R., LoRusso P.M., de Vries E.G. (2024). The Journey of Antibody–Drug Conjugates: Lessons Learned from 40 Years of Development. Cancer Discov..

[8] Drago J.Z., Modi S., Chandarlapaty S. (2021). Unlocking the potential of antibody–drug conjugates for cancer therapy. Nat. Rev. Clin. Oncol..

[9] McPherson M.J., Hobson A.D., Hernandez A., Marvin C.C., Waegell W., Goess C., Oh J.Z., Shi D., Hayes M.E., Wang L. (2024). An anti–TNF–glucocorticoid receptor modulator antibody-drug conjugate is efficacious against immune-mediated inflammatory diseases. Sci. Transl. Med..

[10] Buttgereit F., Aelion J., Rojkovich B., Zubrzycka-Sienkiewicz A., Chen S., Yang Y., Arikan D., D’CUnha R., Pang Y., Kupper H. (2023). Efficacy and Safety of ABBV-3373, a Novel Anti–Tumor Necrosis Factor Glucocorticoid Receptor Modulator Antibody–Drug Conjugate, in Adults with Moderate-to-Severe Rheumatoid Arthritis Despite Methotrexate Therapy: A Randomized, Double-Blind, Active-Controlled Proof-of-Concept Phase IIa Trial. Arthritis Rheumatol..

[11] Thomsen K.L., Møller H.J., Graversen J.H., Magnusson N.E., Moestrup S.K., Vilstrup H., Grønbæk H. (2016). Anti-CD163-dexamethasone conjugate inhibits the acute phase response to lipopolysaccharide in rats. World J. Hepatol..

[12] Svendsen P., Graversen J.H., Etzerodt A., Hager H., Røge R., Grønbæk H., Christensen E.I., Møller H.J., Vilstrup H., Moestrup S.K. (2017). Antibody-Directed Glucocorticoid Targeting to CD163 in M2-type Macrophages Attenuates Fructose-Induced Liver Inflammatory Changes. Mol. Ther.—Methods Clin. Dev..

[13] Brandish P.E., Palmieri A., Antonenko S., Beaumont M., Benso L., Cancilla M., Cheng M., Fayadat-Dilman L., Feng G., Figueroa I. (2018). Development of Anti-CD74 Antibody–Drug Conjugates to Target Glucocorticoids to Immune Cells. Bioconjugate Chem..

[14] Metrangolo V., Engelholm L.H. (2024). Antibody–Drug Conjugates: The Dynamic Evolution from Conventional to Next-Generation Constructs. Cancers.

[15] Harel E.T., Drake P.M., Barfield R.M., Lui I., Farr-Jones S., Veer L.V., Gartner Z.J., Green E.M., Lourenço A.L., Cheng Y. (2019). Antibody-Drug Conjugates Targeting the Urokinase Receptor (uPAR) as a Possible Treatment of Aggressive Breast Cancer. Antibodies.

[16] Junker F., Gordon J., Qureshi O. (2020). Fc Gamma Receptors and Their Role in Antigen Uptake, Presentation, and T Cell Activation. Front. Immunol..

[17] Wilkinson I., Anderson S., Fry J., Julien L.A., Neville D., Qureshi O., Watts G., Hale G. (2021). Fc-engineered antibodies with immune effector functions completely abolished. PLoS ONE.

[18] Balamkundu S., Liu C.-F. (2023). Lysosomal-Cleavable Peptide Linkers in Antibody–Drug Conjugates. Biomedicines.

[19] Sarvothaman S., Undi R.B., Pasupuleti S.R., Gutti U., Gutti R.K. (2015). Apoptosis: Role in myeloid cell development. Blood Res..

[20] Chang J., Wang Y., Shao L., Laberge R.-M., DeMaria M., Campisi J., Janakiraman K., Sharpless N.E., Ding S., Feng W. (2016). Clearance of senescent cells by ABT263 rejuvenates aged hematopoietic stem cells in mice. Nat. Med..

[21] Wang L., Doherty G.A., Judd A.S., Tao Z.F., Hansen T.M., Frey R.R., Song X., Bruncko M., Kunzer A.R., Wang X. (2020). Discovery of A-1331852, a First-in-Class, Potent, and Orally-Bioavailable BCL-X(L) Inhibitor. ACS Med. Chem. Lett..

[22] Hingorani D.V., Allevato M.M., Camargo M.F., Lesperance J., Quraishi M.A., Aguilera J., Franiak-Pietryga I., Scanderbeg D.J., Wang Z., Molinolo A.A. (2022). Monomethyl auristatin antibody and peptide drug conjugates for trimodal cancer chemo-radio-immunotherapy. Nat. Commun..

[23] Vandooren J., Berghmans N., Dillen C., Van Aelst I., Ronsse I., Israel L.L., Rosenberger I., Kreuter J., Lellouche J.-P., Michaeli S. (2013). Intradermal air pouch leukocytosis as an in vivo test for nanoparticles. Int. J. Nanomed..

[24] Opferman J.T. (2007). Apoptosis in the development of the immune system. Cell Death Differ..

[25] D’aRienzo A., Verrazzo A., Pagliuca M., Napolitano F., Parola S., Viggiani M., Caputo R., Puglisi F., Giuliano M., Del Mastro L. (2023). Toxicity profile of antibody-drug conjugates in breast cancer: Practical considerations. eClinicalMedicine.

[26] Liu X., Deng J., Zhang R., Xing J., Wu Y., Chen W., Liang B., Xing D., Xu J., Zhang M. (2023). The clinical development of antibody-drug conjugates for non-small cell lung cancer therapy. Front. Immunol..

[27] Carneiro B.A., Perets R., Dowlati A., LoRusso P., Yonemori K., He L., Munasinghe W., Noorani B., Johnson E.F., Zugazagoitia J. (2023). Mirzotamab clezutoclax as monotherapy and in combination with taxane therapy in relapsed/refractory solid tumors: Dose expansion results. J. Clin. Oncol..

[28] Amor C., Feucht J., Leibold J., Ho Y.-J., Zhu C., Alonso-Curbelo D., Mansilla-Soto J., Boyer J.A., Li X., Giavridis T. (2020). Senolytic CAR T cells reverse senescence-associated pathologies. Nature.

[29] Amor C., Fernández-Maestre I., Chowdhury S., Ho Y.-J., Nadella S., Graham C., Carrasco S.E., Nnuji-John E., Feucht J., Hinterleitner C. (2024). Prophylactic and long-lasting efficacy of senolytic CAR T cells against age-related metabolic dysfunction. Nat. Aging.

[30] Ding Z., Tarlinton D. (2024). Chimeric antigen receptor T cells in the fast lane among autoimmune disease therapies. Clin. Transl. Immunol..

[31] Rubahamya B., Dong S., Thurber G.M. (2024). Clinical translation of antibody drug conjugate dosing in solid tumors from preclinical mouse data. Sci. Adv..

