# Supplementary material for: uPAR-Targeting Cytotoxic Antibody–Drug Conjugates Selectively Deplete Proinflammatory Myeloid Cells for Autoimmune Indications
Source: Cells. 2026 Apr 29;15(9):803. doi: 10.3390/cells15090803 (PMC13162639; doi:10.3390/cells15090803)

Supplementary Figure Legend

Supplementary Figure 1 (A). The profiling of 2G10-hIgG1-LALADS antibodies by analytical size-exclusion chromatography. The analysis, monitored at an absorbance of 280 nm, revealed that the antibody sample was 96.4% monomeric, a good purity for subsequent conjugation. (B-C) The chemical structure of ABT-263 linker-payload (B) and A-1331852 linker-payload (C).

Supplementary Figure 2 (A). The percentage of relative cell viability of mouse uPAR-overexpressing CHO-K1 cells treated with Saporin, Isotype control rat IgG, anti-uPAR mAb-028 and mAb-006 for 3 days. The rat IgG serves as a negative control. mAb-028 and mAb-006 showed dose-response killing, indicative of antibody internalization. (B). The cell viability of proinflammatory mouse peritoneal macrophages stimulated with GM-CSF and TNFα treated with isotype-control, anti-uPAR mAb006 ADCs, or the free payloads (ABT-263) for 4 days. (C-D) The dose-response killing curves of ABT-263 and A-1331852 in human inflammatory macrophages stimulated with TNFα and PGE_2 (C) or in mouse peritoneal macrophages stimulated with GM-CSF and TNFα (D). (E) The chemical structure of the MMAF linker-payload: Mc-Val-Cit-PAB-MMAF. (F) Geometric mean fluorescence intensity (GeoMFI) of cell surface uPAR staining by mAb029 of mouse peritoneal macrophages treated with different concentrations of mAb-028-MMAF or Isotype Control-MMAF conjugates.

Supplementary Figure 3 (A). A representative FACS plot of cell surface uPAR expression from indicated immune subsets. (B). The body weight of mice treated with PBS, isotype control-MMAF, or uPAR-MMAF.

Supplementary Figure 1

A.

2G10-hIgG1-LALADS

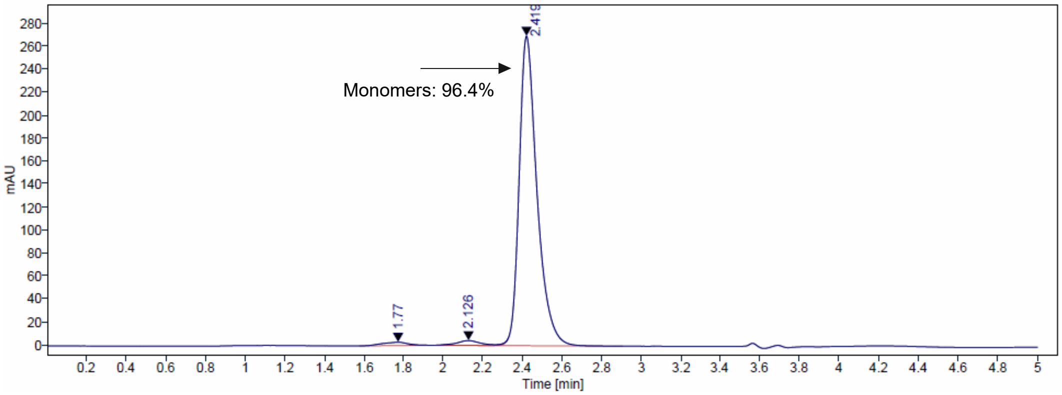

B.

The chemical structure of ABT-263-linker-payload

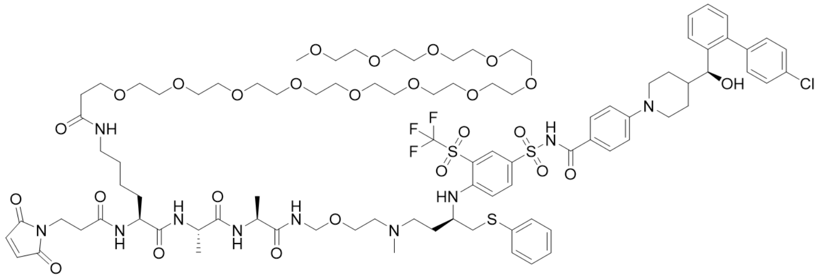

C.

The chemical structure of A-1331852-linker-payload

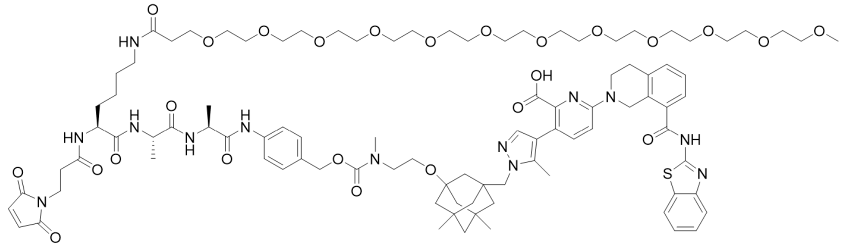

Supplementary Figure 2

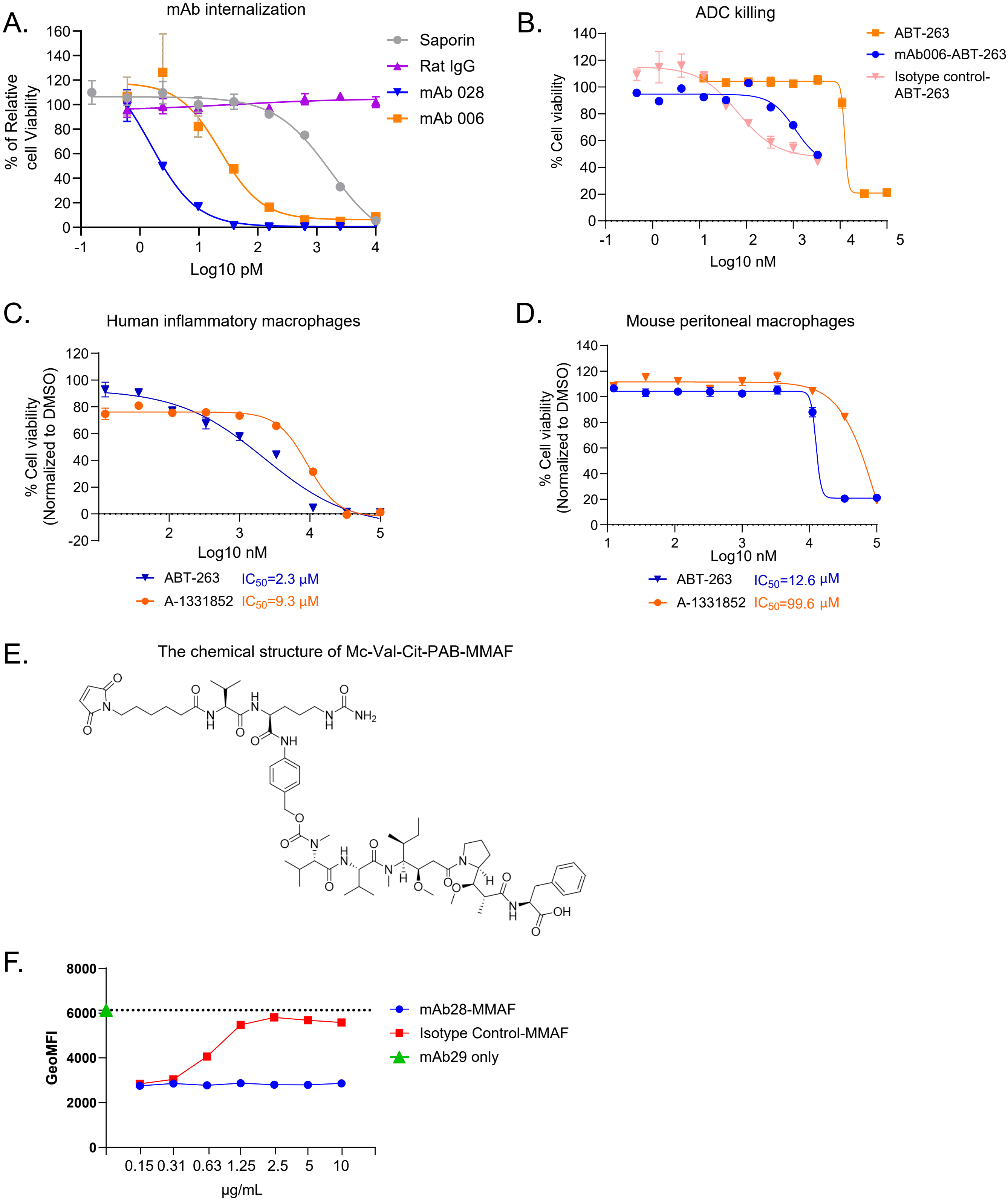

Supplementary Figure 3

A.

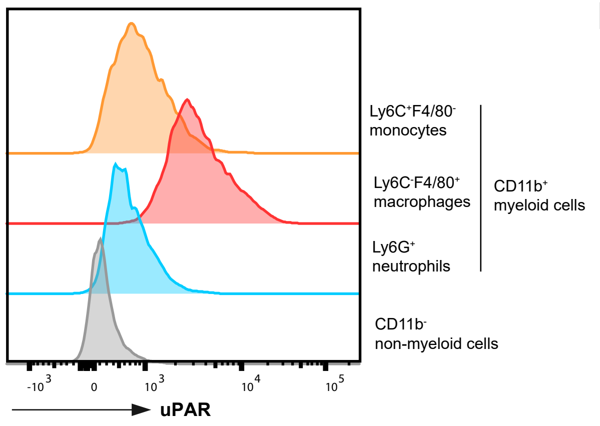

B.

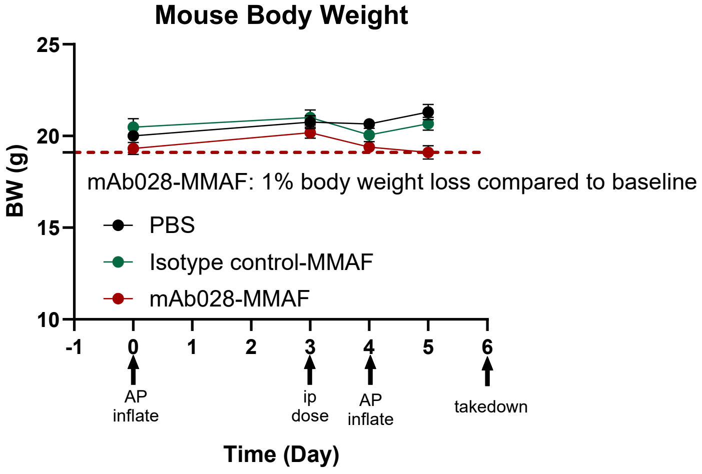

Supplement: Supplementary file 1 [file cells-15-00803-s001.zip › supplementary combined.pdf]
